# Effects of three IL-15 variants on NCI-H446 cell proliferation and expression of cell cycle regulatory molecules

**DOI:** 10.18632/oncotarget.22550

**Published:** 2017-11-20

**Authors:** Jun-Ying Ding, Zhi-Hua Wang, Zheng-Zheng Zhang, Xu-Ran Cui, Yan-Ying Hong, Qing-Quan Liu

**Affiliations:** ^1^ Beijing Key Laboratory of Basic Study on Traditional Chinese Medicine (TCM) Infectious Diseases, Beijing Hospital of TCM, Capital Medical University, Beijing Institute of TCM, Beijing, China; ^2^ Hebei Key Laboratory of Metabolic Disease, Hebei General Hospital, Shijiazhuang, China; ^3^ Department of Immunology and Key Laboratory of Immune Mechanism and Intervention on Serious Disease, Hebei Medical University, Shijiazhuang, China

**Keywords:** interleukin 15, NCI-H446 cells, cell proliferation, cell cycle, cell cycle regulatory molecule

## Abstract

Interleukin 15 (IL-15) is a cytokine exhibiting antitumor characteristic similar to that of IL-2. However, in human tissues and cells, IL-15 expression and secretion is very limited, suggesting IL-15 functions mainly intracellularly. In the present study, we assessed the effects of transfecting NCI-H446 small cell lung cancer cells with genes encoding three IL-15 variants: prototypical IL-15, mature IL-15 peptide, and modified IL-15 in which the IL-2 signal peptide is substituted for the native signal peptide. NCI-H446 cells transfected with empty plasmid served as the control group. We found that IL-15 transfection effectively inhibited NCI-H446 cell proliferation and arrested cell cycle progression, with the modified IL-15 carrying the IL-2 signal peptide exerting the greatest effect. Consistent with those findings, expression each of the three IL-15 variants reduced growth of NCI-H446 xenograph tumors, and the modified IL-15 again showed the greatest effect. In addition, IL-15 expression led to down-regulation of the positive cell cycle regulators cyclin E and CDK2 and up-regulation of the negative cycle regulators p21 and Rb. These findings suggest IL-15 acts as a tumor suppressor that inhibits tumor cell proliferation by inducing cell cycle arrest.

## INTRODUCTION

Among the new approaches to treating various cancers is gene therapy, in which the gene encoding an anti-tumor cytokine is transfected into the tumor cells [1, 2]. For example, the cytokine IL-15 exhibits biological activity similar of IL-2 in that it enhances proliferation of CD8+CTL and natural killer (NK) cells, which in turn kill tumor cells [3–9].

IL-15 mRNA is readily detected in many kinds of cells and tissues, but secretion of the corresponding protein is rarely detected, which suggests IL-15 protein expression is controlled by posttranscriptional mechanisms – i.e., at the level of protein translation and intracellular trafficking [10–18]. Alternatively, other data suggest IL-15 carries out its biological functions mainly intracellularly [19]. It was observed in nude mice inoculated with tumor cells that transfecting the cells with a gene encoding prototypical IL-15 did not yield a promising anti-tumor effect. It was suggested that the long signal peptide expressed with IL-15 acts as an inhibitor to IL-15 secretion; however, transcription of a modified IL-15 gene lacking the signal peptide did not substantially increase IL-15 secretion [20].

In an earlier study, we observed that transfection with genes encoding different IL-15 variants genes led to obvious decreases in the proliferation of NCI-H446 small cell lung cancer cells. One of the malignant characteristics of these tumor cells is accelerated proliferation resulting in part from cell cycle dysregulation. Cell proliferation is controlled in part by cell cycle-positive/negative regulatory molecules, including cell cycle protein (cyclin), cyclin-dependent kinase (CDK), p21 and retinoblastoma (Rb) [21, 22]. In the present study, we examined the effects of transfecting genes encoding different IL-15 variants on the cell proliferation and the expression of cell cycle-positive/negative regulatory molecules in NCI-H446 cells were examined, and the tentative anti-tumor activities of transfected genes were observed *in vivo*.

## RESULTS

### IL-15 gene transfection inhibited NCI-H446 cell proliferation

**Figure 1:**
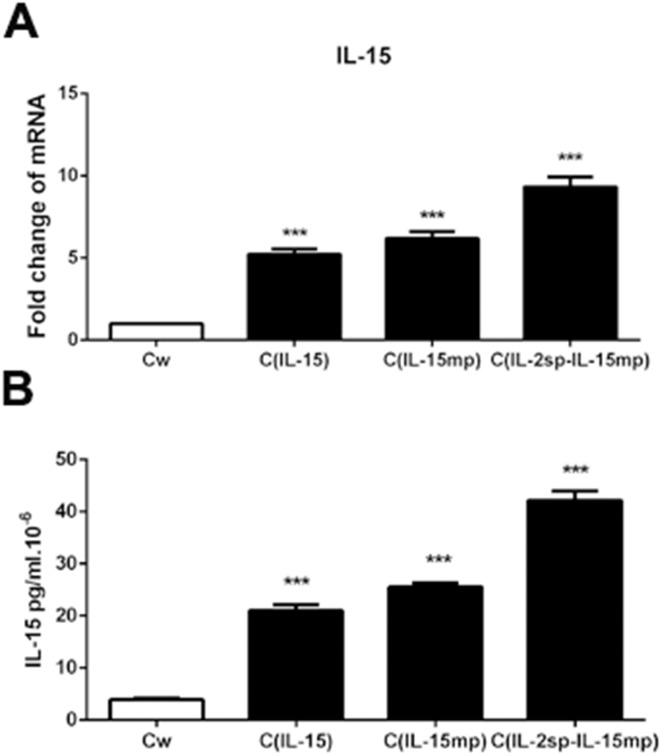
Transfection of genes encoding three IL-15 variants into NCI-H446 cells Following transfection, NCI-H446 cells prototypical IL-15 (C_IL-15_ group), mature IL-15 peptide (C_IL-15mp_ group), modified IL-15 in which the native signal peptide was replaced with the IL-2 signal peptide (C_IL-2sp-IL-15mp_ group), and wild prototype NCI-H446 Cell (Cw) transfected with control plasmid as control group. **(A)** IL-15 mRNA levels determined using RT-qPCR. **(B)** IL-15 protein levels determined using an ELISA (^***^P<0.001).

We initially transfected NCI-H446 cells with genes encoding three different IL-15 variants: the C_IL-15_ group was transfected with the IL-15 prototype, the C_IL-15mp_ group was transfected with mature IL-15 peptide, and the C_IL-2sp-IL-15mp_ group was transfected with a modified IL-15 in which the IL-15 signal peptide was replaced with IL-2 signal peptide. As a control, NCI-H446 cells were transfected with an empty plasmid. Then using real time quantitative PCR and ELISAs, we observed that transfection with the different IL-15 variants increased expression of IL-15 mRNA and secretion of IL-15 protein from NCI-H466 cells to different degrees (Figure 1A and 1B).

**Figure 2:**
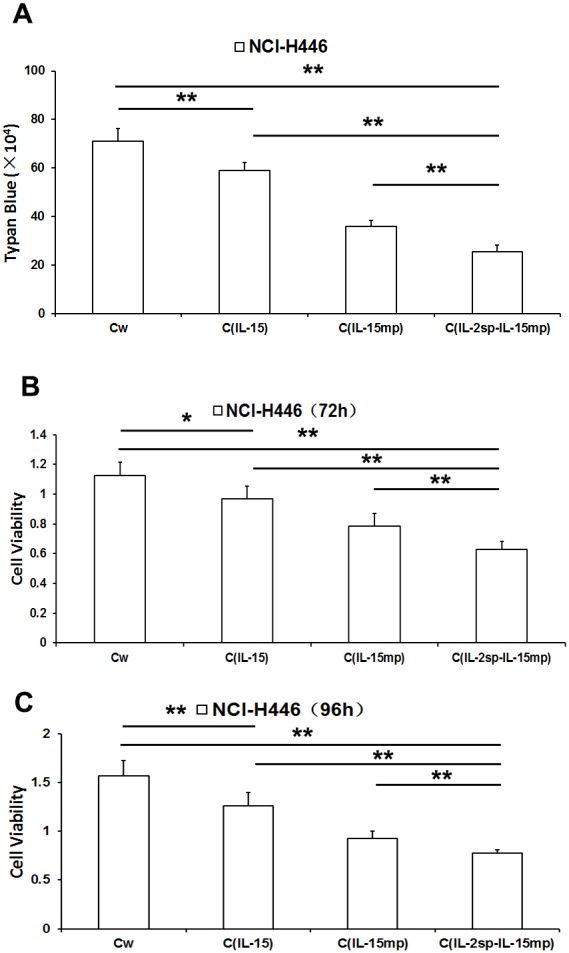
Effects of three IL-15 variants on NCI-H446 cell proliferation **(A)** Living NCI-H446 cell counts determined based on trypan blue dye exclusion. **(B)** NCI-H446 cell proliferation assessed using MTT assay at 72h. **(C)** NCI-H446 cell proliferation assessed using MTT assay at 96h. (^*^P<0.05, ^**^P<0.01).

As shown in Figure 2A, there were significantly fewer surviving cells in C_IL-15_, C_IL-15mp_ and C_IL-2sp-IL-15mp_ groups than in the control group, with the greatest reduction in survival in the C_IL-2sp-IL-15mp_ group. Correspondingly, MTT assays revealed that while cell proliferation was lower in all three transfectant groups than in the control group, the greatest decrease in proliferation was in the C_IL-2sp-IL-15mp_ group (Figure 2B and 2C). These results suggest transfection with prototypical IL-15 effectively inhibited the NCI-H446 cell proliferation, but that effect was enhanced by transfection with modified IL-15 carrying the IL-2 signal peptide.

### IL-15 transfection hindered NCI-H446 cell cycling

**Figure 3:**
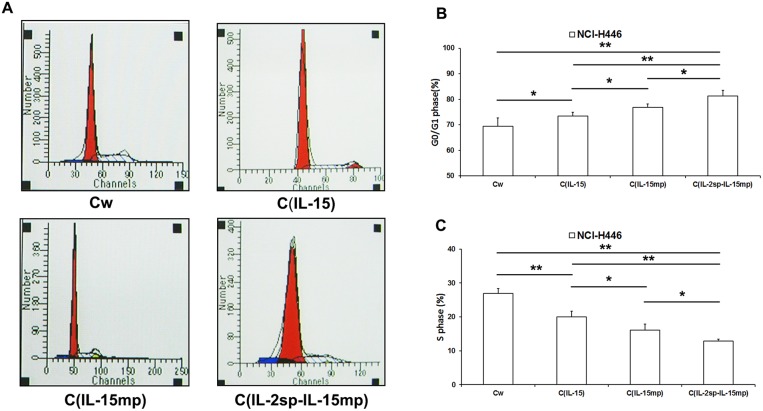
Effects of the three IL-15 variants on NCI-H446 cell cycling **(A)** The cell cycle of NCI-H446 cells was examined using PI staining and flow cytometry. **(B)** The ratio of G0/G1 phase in NCI-H446 cells was caculated according to the data from flow cytometry. **(C)** The ratio of S phase in NCI-H446 cells was caculated according to the data from flow cytometry. (^*^P<0.05, ^**^P<0.01).

The cell cycle is a key determinant controlling cell proliferation. We therefore tested the effects of expressing the different IL-15 variants on the NCI-H446 cell cycle. We observed that the cell fractions in G0/G1 or S phase were significantly higher in the three transfectant groups than in the control group (Figure 3A-3C). Moreover, NCI-H446 cells in the C_IL-2sp-IL-15mp_ group exhibited the greatest potential for cell cycle arrest. These results suggest that expression of prototypical IL-15 effectively inhibited NCI-H446 cell cycling, and the IL-2 signal peptide strengthened the inhibitory effect of IL-15 on the cell cycle.

### Effect on the expression of cell cycle-positive regulatory molecules

**Figure 4:**
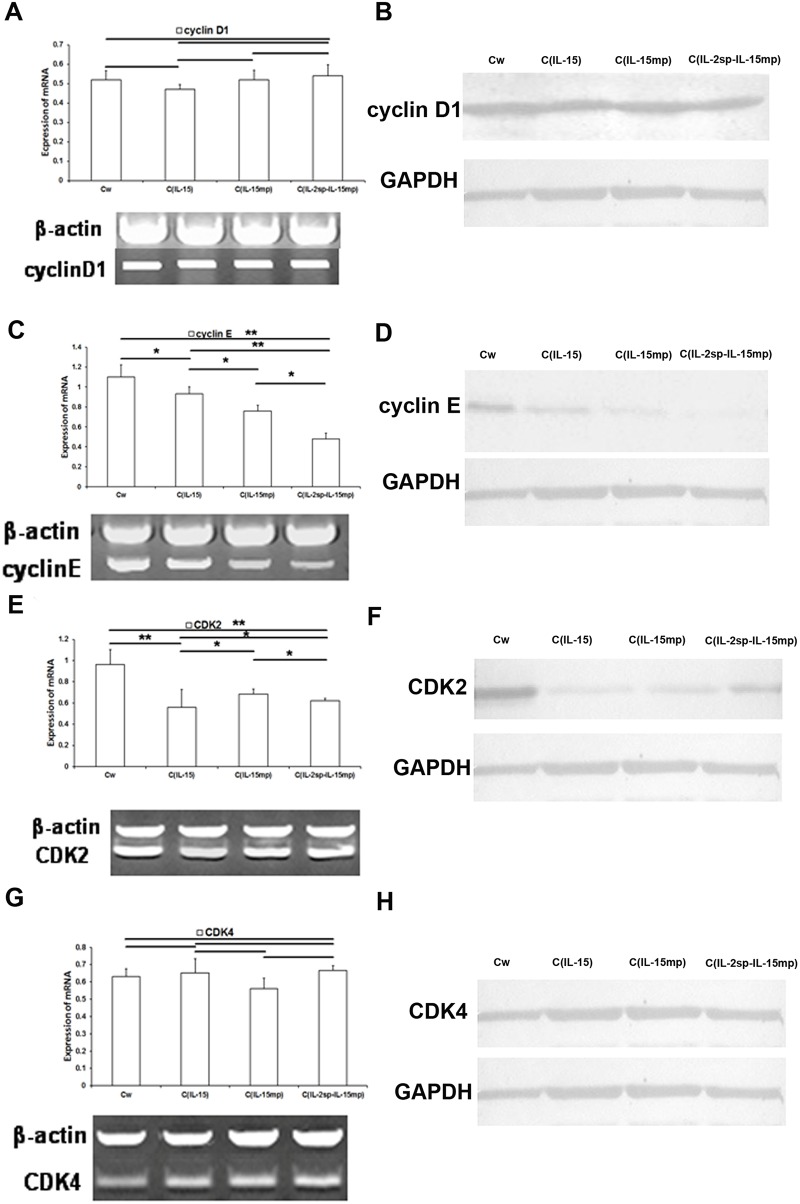
Effects of the three IL-15 variants on positive regulators of the cell cycle in NCI-H446 cells **(A, C, E** and **G)** Levels of cyclinD1, cyclin E, CDK2 and CDK4 mRNAs in NCI-H446 cells determined using RT-qPCR. **(B, D, F** and **H)** Level of cyclinD1, cyclin E, CDK2 and CDK4 protein in NCI-H446 cells determined based on western blotting (^*^P<0.05, ^**^P<0.01).

When we then examined the effects of expressing the three IL-15 variants on cell cycle-positive regulatory molecules, we found that cyclin D1 expression was unaffected, whereas cyclin E expression was significantly lower in the transfectant groups than in the controlgroup (Figure 4A and 4B). In addition, the magnitude of the suppression of cyclin E expression varied among the C_IL-15_, C_IL-15mp_ and C_IL-2sp-IL-15mp_ groups (Figure 4C and 4D). Overall, however, it appears IL-15 gene transfection decreases cyclin E expression.

Similarly, expression of CDK2 mRNA and protein was lower in the C_IL-15_, C_IL-15mp_ and C_IL-2sp-IL-15mp_ groups than in the control group (Figure 4E and 4F). By contrast CDK4 levels did not significantly differ among the four groups (Figure 4G and 4H).

### Effect on the expression of cell cycle-negative regulatory molecules

**Figure 5:**
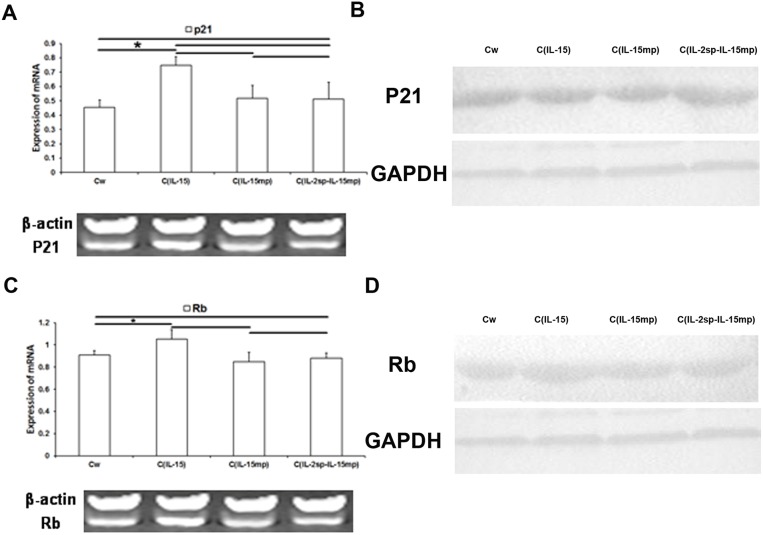
Effects of the three IL-15 variants on negative regulators of the cell cycle in NCI-H446 cells **(A, C)** Levels of p21 and Rb mRNA in NCI-H446 cells determined using RT-qPCR. **(B, D)** Levels of p21 and Rb protein in NCI-H446 cells determined based on western blotting (^*^P<0.05, ^**^P<0.01).

Because p21 and Rb reportedly play key roles in the cell cycle [23], we examined the effect of expressing each of the three IL-15 variants on p21 and Rb expression. Levels of p21 mRNA and protein were significantly higher in the C_IL-15_ than the control group. On the other hand, levels of p21 in the C_IL-15mp_ and C_IL-2sp-IL-15mp_ groups did not differ from control (Figure 5A and 5B). This suggests prototypical IL-15 gene transfection could promote p21 expression, but IL-15 mature peptide gene and the IL-15 modified gene couldn’t.

As with p21, levels of Rb expression in the C_IL-15_ group were significantly higher than control, whereas Rb expression in the C_IL-15mp_ and C_IL-2sp-IL-15mp_ groups did not differ from control (Figure 5C and 5D). These results suggest expression of prototypical IL-15 led to increases in Rb expression in NCI-H446 cells, though mature IL-15 and the modified IL-15 had no effect.

### The anti-tumor potential of IL-15 in NCI-H446 cells

**Figure 6:**
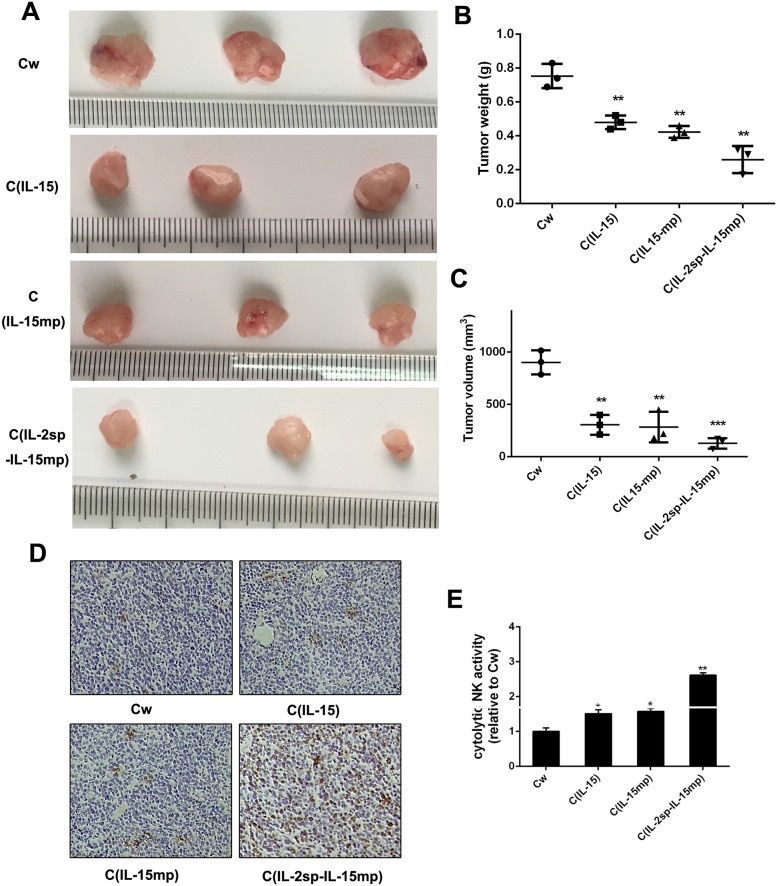
Anti-tumor potential of IL-15 in NCI-H446 cells Tumorigenicity was detected *in vivo*. Photographs were taken 4 weeks after subcutaneous injection. **(A)** Photographs of representative tumors. **(B, C)** Quantitative analysis of tumor weights and volumes in the indicated groups. Data are presented as the means (±SD) of three independent experiments (^**^P < 0.01, ^***^P<0.001). **(D, E)** Infiltrating cells were isolated from tumor tissues and stained with anti-NK cell antibodies (^*^P<0.05, ^**^P<0.01).

To assess the anti-tumor activity of IL-15 *in vivo*, equal volumes of the three NCI-H466 transfectants were injected subcutaneously into mice. The apparent tumorigenicity of cells from the C_IL-15_, C_IL-15mp_ and C_IL-2sp-IL-15mp_ groups was lower than that of control NCI-H466 cells (Figure 6A-6C). In particular, tumors formed by cells from the C_IL-2sp-IL-15mp_ group exhibited the smallest weights and volumes. Thus IL-15 appears to effectively reduce the tumorigenicity of NCI-H466 small cell lung cancer cells.

It is known that IL-15 enhances NK cell activity [24], which suggests NK cells may play an important role in the observed tumor suppression. Consistent with that idea, greater NK cell activity was detected in tumors composed of C_IL-15_, C_IL-15mp_ or C_IL-2sp-IL-15mp_ transfectants than tumors composed of control NCI-H446 cells (Figure 6D and 6E). This suggests IL-15 expression may inhibit NCI-H446 tumorigenicity by stimulating NK cell activity.

## DISCUSSION

IL-15 was a cytokine with biological activity similar to that of IL-2. For example, like IL-2 it enhances CD8+CTL and NK cell proliferation and tumor cell killing. As such, IL-15 is one of the most promising molecules for antitumor immunotherapy. On the other hand, transfecting tumor cells with prototypical IL-15 before injecting them into nude mice did achieve a promising antitumor effect. Much research has focused on the ability of IL-15 to inhibiting tumor cell proliferation *in vivo* [25], but the potential mechanism underlying the antitumor activity of IL-15 also involves up-regulating NK cell and CTL activity. Consistent with that idea, we found that modifying IL-15 with the IL-2 signal peptide enhanced IL-15 secretion as well as its ability to induce cell cycle arrest in NCI-H446 small cell lung cancer cells and increase NK cell antitumor activity.

Molecules affecting cell proliferation include those that exert a stimulatory effect, such as cyclin and CDK [26–29], and those that exert an inhibitory effect, such as p21 and Rb [21–22]. Rb is a tumor suppressor gene whose product acts as a negative regulator of the cell cycle, and its function loss can directly result in tumor development. Cyclin D1 and cyclin E are overexpressed in some tumor cells, where they induce cell cycle progression from G1 to S phase. Inhibiting their expression can induce cell cycle arrest in G0/G1 phase, thereby suppressing cell proliferation [27–29]. p21 binds to and inhibits the activities of CDK2 and CDK4. p21 also inhibits the activity of PCNA by inhibiting the DNA synthesis directly. In the present study, we found that the inhibitory effect on the cell cycle in the C_IL-2sp-IL-15mp_ group was likely mediated by cyclin E and CDK2, not cyclin D1 or CDK4. However, we also found that whereas prototypical IL-15 increased expression of both p21 and Rb, cells in the C_IL-2sp-IL-15mp_ group did not exhibit a significant effect on p21 or Rb expression.

In sum, our findings suggest IL-15 suppresses NCI-H446 small cell lung cancer cell proliferation through cell cycle arrest mediated by cyclin E and CDK2. In addition, IL-15 exerts antitumor effects through stimulation of NK cell activity.

## MATERIALS AND METHODS

### Cell culture

NCI-H446 cells were cultured in Dulbecco's Modified Eagle Medium (DMEM; Invitrogen Gibco Cell Culture Products, Carlsbad, CA) with 10% fetal bovine serum (FBS; Invitrogen, Carlsbad, CA), 50 units of penicillin, 50 μg/mL gentamicin, 2.5 μg/mL amphotericin B, 1% glutamine, 2% HEPES at 37°C in a humidified atmosphere containing 5% CO_2_.

### Plasmid construction and gene transfection

Genes encoding prototypical IL-15, mature IL-15 peptide, and modified IL-15 (IL-2 signal peptide linked to mature IL-15) were cloned into a plasmid from Gateway Cloning System (Invitrogen, Carlsbad, CA). The sequences were designed using Oligo engine software.

NCI-H446 cells (1×10^6^ cells/dish) were plated in 10-cm Petri dishes and cultured to 60% confluence. The cells were then transfected using a Lipofectamine 2000 kit (Invitrogen, cat. No. 11668-019) and harvested 48 h after transfection. The three transfectant groups included the C_IL-15_ group transfected with prototypical IL-15, the C_IL-15mp_ group transfected with the mature IL-15 peptide, and the C_IL-2sp-IL-15mp_ group transfected with modified IL-15. Transfection efficiency was assessed using real time-PCR and ELISAs.

### RNA extraction and real-time quantitative PCR (qPCR)

Total RNA extraction, complementary DNA (cDNA) synthesis, and qPCR were performed as described previously [15]. Total mRNA was extracted from NCI-H446 cells using an RNeasy Mini Kit (Qiagen, Valencia, CA) according to the manufacturer's protocol. The RNA was used to generate first strand cDNA using random primers and Super Script II reverse transcriptase (Invitrogen). Real-time PCR was performed using as SYBR Prime Script RT-PCR Kit (Takara, Dalian). GAPDH expression was detected as an internal control. The first step of the PCR was incubation at 50°C for 2 min and then at 95°C for 10 min. This was followed by 40 cycles of 95°C for 15 s and 60°C for 60 s in a Mx4000 system from Stratagene (La Jolla, CA). The primer sequences of qPCR are shown in Table 1.

**Table 1:** Sequences of RT-PCR oligonucleotide primers

Gene		Sequence (5’ → 3’)
cyclin D1 (165bp)	F	AAGATCGTCGCCACCTGGA
	R	CTTAGAGGCCACGAACATGCAA
cyclin E (446bp)	F	ATCCCCACACCTGACAAAGAAG
	R	CCTGAACAAGCTCCATCTGTCA
CDK2 (317bp)	F	GCTTTCTGCCATTCTCATCG
	R	GTCCCCAGAGTCCGAAAGAT
CDK4 (488bp)	F	ATTGGTGTCGGTGCCTATGG
	R	ACGGGTGTAAGTGCCATCTG
GAPDH	F	TGCCAAATATGATGACATCAAGAA
	R	GGAGTGGGTGTCGCTGTTG

### Enzyme-linked immunosorbent assay (ELISA)

Cytokine concentrations in culture supernatants were measured using a human IL-15 ELISA kit (BD Bioscience, Franklin Lakes, NJ).

### Cell viability assay

NCI-H446 cells were seeded into a 96-well plate and incubated for 24 h. The cells were then treated with various concentrations of cisplatin, after which MTT assays were performed. Twenty microliters of 5 mg/ml MTT in PBS were added to each well and incubated for 4 h at 37°C. The formazan crystals formed were dissolved in 200 μl of DMSO, and the optical density of the solution at 570 nm (OD_570_) was determined. Each assay was performed three times, and the average results were calculated.

### Cell cycle analysis

For cell cycle analysis, aliquots (1×10^6^) of cells were fixed overnight in 70% ethanol at 4°C. The cells were then washed in PBS and stained with propidium iodide (PI; Sigma, Cat. P4170). After 30 min at room temperature in the dark, the cells were filtered through 40 μm diameter mesh to remove clumps of nuclei. Flow cytometry (BD FACS Aria, Becton Dickinson) was the used to identify cells in the sub-G1, G0-G1, S or G2-M phases. The sub-G1 nuclei in the populations were deemed apoptotic.

### Protein extraction and western blot (WB)

Soluble proteins were extracted from cells, and western blot analysis was performed as described previously [14]. Specific antibodies for cyclin D1 (1:1000 dilution, Santa Cruz, Inc.), cyclin E (1:2000 dilution, Novus Biologicals, Inc.) and CDK2 (1:2000 dilution, Novus Biologicals, Inc.) were used as primary antibodies. Horseradish peroxidase-conjugated goat anti-rabbit IgG was the secondary antibody (R&D Systems, Cat. BAF008). GAPDH served as an internal control.

### Animal model

Cells were injected subcutaneously into the left back of six-week-old BALB/c nude mice at 1×10^6^cells/injection site. At the end of 4 weeks, mice were sacrificed. The weight and volume of the tumor was detected.

### NK cell activity assay

NK cell cytolytic activity was assessed using mouse splenocyte effectors and YAC-1 cell targets at different effector/target (E/T) ratios. Target cells were labeled with ^51^Cr. The percent specific lysis was calculated using the following formula: percent cytotoxicity = [(experimental release – spontaneous release by effector and target)/(maximal release – spontaneous release)] × 100.

### Statistical analysis

All data were expressed as the mean ± SEM from at least three separate experiments. Data were analyzed using ANOVA for comparisons among multiple groups or using Student t-test for comparison between two groups. All analyses were performed using the Microsoft Excel Analysis Tool Pak (Microsoft, Redmond, WA). Values of P<0.05 were considered significant.
